# Disease, Drugs and Dysbiosis: Understanding Microbial Signatures in Metabolic Disease and Medical Interventions

**DOI:** 10.3390/microorganisms8091381

**Published:** 2020-09-09

**Authors:** Ceri Proffitt, Gholamreza Bidkhori, David Moyes, Saeed Shoaie

**Affiliations:** 1 Centre for Host–Microbiome Interactions, Faculty of Dentistry, Oral & Craniofacial Sciences, King’s College London, London SE1 9RT, UK; gholamreza.bidkhori@kcl.ac.uk (G.B.); david.moyes@kcl.ac.uk (D.M.); 2 Science for Life Laboratory, Department of Protein Science, KTH Royal Institute of Technology, 114 17 Stockholm, Sweden

**Keywords:** metabolic diseases, metagenomics, systems biology, dysbiosis, gut microbiota

## Abstract

Since the discovery of the potential role for the gut microbiota in health and disease, many studies have gone on to report its impact in various pathologies. These studies have fuelled interest in the microbiome as a potential new target for treating disease Here, we reviewed the key metabolic diseases, obesity, type 2 diabetes and atherosclerosis and the role of the microbiome in their pathogenesis. In particular, we will discuss disease associated microbial dysbiosis; the shift in the microbiome caused by medical interventions and the altered metabolite levels between diseases and interventions. The microbial dysbiosis seen was compared between diseases including Crohn’s disease and ulcerative colitis, non-alcoholic fatty liver disease, liver cirrhosis and neurodegenerative diseases, Alzheimer’s and Parkinson’s. This review highlights the commonalities and differences in dysbiosis of the gut between diseases, along with metabolite levels in metabolic disease vs. the levels reported after an intervention. We identify the need for further analysis using systems biology approaches and discuss the potential need for treatments to consider their impact on the microbiome.

## 1. Introduction

Microbiota is the collective term for the ecosystem of microbial organisms living in a particular habitat. These ecosystems occupy a wide variety of habitats, including environmental regions such as coastal marine environments [1], animal environments (e.g., murine gut [2]) and human niches such as the skin [3], blood [4], mouth [5], nasal cavity [6] and gut [7]. The largest and most studied microbiome in humans is in the gut [8] with the oral cavity a close second. The gut microbiome community is made up of more microbial cells than there are human cells in the body, giving rise to hypotheses that it should be treated as a human organ [9]. The gut microbiome is impacted by several different sociodemographic factors such as age [10], geography [11], diet [12] and exercise [13]. It constantly evolves, growing and adapting depending on environmental factors and bacterial relationships. Bacterial species within these communities interact on a variety of levels via mutualistic, commensal, competitive or other relationships. These communities are far from inert, having an impact on their hosts in a variety of ways. As we have evolved, so too have these communities, developing a population that exists in parallel to their host. We now believe that the microbiome and factors affecting it are highly significant in human health [12]. These diverse ecosystems have various impacts on host physiology, such as modulation of the immune system by short chain fatty acid production (known to regulate T cell numbers) [14]. Further, these communities provide protection from pathogenic invasion through niche competition [15] and can aid in food digestion, in particular the breakdown of fibre [16].

However, when the environment of a habitat is disturbed this can alter the composition of microbial species in the eco-system [17]. These alterations can result in an imbalance between the host and microbial community or a reduction in microbial diversity in the gut known as dysbiosis. Many previous studies show the impact gut dysbiosis has in human diseases, such as type 2 diabetes [18], atherosclerotic cardiovascular disease (ACVD) [19], obesity [20] and liver cirrhosis [21]. 

As well as environmental changes, dysbiosis in the gut microbial communities can be caused by medical intervention such as drug use and bariatric surgery [22]. Antibiotics have been shown to profoundly alter the microbiota, supressing growth/presence of some species, thereby causing a loss of diversity [23]. Proton pump inhibitors (PPIs) used for inhibiting stomach acid production have been associated with high levels of oral bacteria in the gut, potentially by degrading the stomach acid barrier to microbial colonisation [24]. Metformin has been associated with reducing local inflammation and lipid absorption which can then alter the microbiome to significantly increase *Escherichia* species [25]. Gastric bypass surgeries (GBs) are a highly effective procedure for morbidly obese patients, who commonly suffer from obesity-related co-morbidities. With their impact on gastric function and nutrient passage, these procedures can also cause disruption to gut microbial composition. GBs have a direct effect on the microbiome, and recent studies have indicated that changes induced in the microbiome may facilitate some of the positive effects of surgery [26]. It is important to understand mechanisms behind the behaviour in the microbiome of metabolic disease and drug interventions. This will allow for a more targeted approach to treating disease via the microbiome. Here we review the dysbiosis associated with metabolic diseases, the effect medication has on the microbiota and assess the changes caused by a gastric band procedure. 

## 2. Alterations in the Microbiome Associated with Disease

The three most common metabolic diseases are obesity, type 2 diabetes (T2D) and ACVD. Obesity, defined by having a body mass index (BMI) of more than 30, is a growing concern globally for health and wellness [27], and diagnosed cases of T2D are of equal concern. T2D, caused by insulin resistance, means the blood sugar levels become too high. Both these conditions could be impacted by diet, genetics and environment [28]. ACVD is a coronary condition that is caused by formation of a plaque (comprised of fat, cholesterol, calcium and other substances such as macrophages and fibroblasts) within the arteries that supply the heart. In a 2016 World Health Organisation (WHO) report an estimated 422 million adults worldwide suffered from T2D, while more than 1 in 10 adults were obese [29]. Likewise, cardiovascular disease is the number one cause of mortality worldwide [30]. These diseases are associated with sex, age, geography and other factors [31]; in particular diet and genetics are both important influences [28]. Importantly, these diseases are common comorbidities for each other [32]. These diseases are end points to many other conditions which belong to the category of metabolic syndrome. They are the most serious stages of disease which can lead to hospitalisation and surgery. However, conditions such as hypertension, which are symptomless, can go unnoticed for years [33] and are often intertwined with diseases such as T2D and obesity; with up to 75% of diabetic patients suffering from hypertension [34,35]. Notably, even in these common symptomless comorbidities, there are already effects on the gut microbiome composition [36].

Obesity, T2D and ACVD have been linked to dysbiosis in the gut microbiome. For each disease, the metagenomic signatures varied. However, there are key genera which are found to be significantly increased or decreased when compared to the matched controls in more than one study (Supplementary Table S1). Across studies we have focused on reporting the genus and phylum level bacterial signatures, and summarise the bacterial signature overlap between the different diseases.

**Figure 1:**
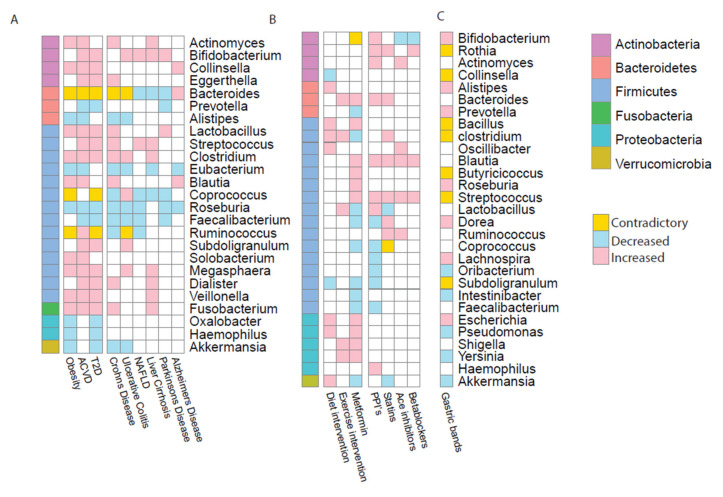
Genus signatures observed throughout the diseases and medical interventions. (**A**) Specific genera signatures observed to be statistically significant in metabolic diseases compared to healthy match controls; including obesity, type 2 diabetes (T2D) and atherosclerosis (ACVD) and also observed in Crohn’s disease, ulcerative colitis, non-alcoholic fatty liver disease (NAFLD), liver cirrhosis, Parkinson’s disease and Alzheimer’s disease. (**B**) Specific genera signatures observed in medical interventions and drug treatments including diet and exercise interventions, metformin, proton pump inhibitors (PPIs), statins, angiotensin-converting-enzyme (ACE) inhibitors and betablockers. (**C**) Specific genera signatures observed in medical interventions also observed in gastric band surgery, including laparoscopic adjustable Gastric band, vertical sleeve gastrectomy and gastric roux-en-y bypass. For all panels blue indicates the genus is reported as decreased in disease than in healthy controls, red indicates the genus is reported as increased in disease than in healthy controls. Yellow shows the genus has been reported increased and decreased in different studies.

Studies comparing patients of these diseases with healthy matched controls showed dysbiotic bacterial signatures within the gut microbiome [18,20,37,38,39]. For each of these diseases, there were alterations at genus and phylum level in the microbiome. All three metabolic diseases showed an increase in Actinobacteria and a decrease in Bacteroidetes. Results also revealed six genera increased in obesity, T2D and ACVD (*Clostridium*, *Collinsella*, *Fusobacterium*, *Lactobacillus*, *Megasphaera* and *Veillonella*); while *Roseburia* was the only genus shown to be consistently decreased across all three metabolic diseases (Figure 1A) [19,20,37,38,39]. 

Metabolic diseases are known to be co-morbidities of other ailments, hence we compared studies of metagenomic analyses from inflammatory bowel disease (IBD), liver diseases and neurodegenerative diseases; as all these diseases could be associated to the gut microbiome. IBD is the term used to cover ulcerative colitis (UC) and Crohn’s disease (CD). Both affect the gut though inappropriate, unrestrained inflammation and cause ulcers in the intestine [40]. When comparing IBD patients with healthy controls, bacterial signatures showed an overlap with metabolic disease signatures. Notably, Actinobacteria (including *Actinomyces*, *Bifidobacterium* and *Eggerthella)* are reported to be enriched in IBD- and MD-patients whilst five genera were decreased in abundance in both IBD diseases and metabolic diseases (*Alistipes*, *Eubacterium*, *Roseburia*, *Feacalibacterium* and *Akkermanisa*) [37,41,42,43,44]. These are known short chain fatty acid producers which are beneficial to health, hence a reduction in these genera could lead to poor health. 

Obesity and T2D are both common comorbidities of liver problems with 80–90% of obese patients and 70% of T2D patients suffering with non-alcoholic fatty liver disease (NAFLD) [45]. NAFLD NAFLD is defined by the accumulation of fat in the liver in those who consume little to no alcohol. NAFLD can in some cases lead to inflammation and scaring of the liver tissue. However, liver cirrhosis is a liver disease which involves scarring of the organ tissue leading to malfunction, often caused by poor diet with excessive alcohol consumption. Similarly to IBD, when comparing liver failure bacterial taxonomic signatures with metabolic diseases there were some concordant results. As for other diseases, Actinobacteria are increased in abundance in cirrhosis [41]. Liver cirrhosis also showed consistent enrichment of genera in the Firmicutes phylum, including *Streprococcus*, *Clostridium*, *Megasphaera*, *Dialister* and *Fusobacterium* which were also enriched in MDs [37,46,47,48]. It is not just gut-associated ailments that are impacted by the microbiome. Increasingly, neurodegenerative diseases have been associated with the gut microbiome via the gut–brain-axis. They have also been increasingly associated with metabolic disease, in particular obesity [49]. Alzheimer’s, the most common neurodegenerative disease, results in an unrepairable loss of neurons [50] whilst Parkinson’s disease, another common neurodegenerative disease, is commonly characterised by tremors, rigidity and instability [50]. Both the conditions show decreased numbers of genera from the phylum Firmicutes, in contrast to liver disease. However, the few genera present show enrichment, discordant with other diseases [51,52,53,54]. 

In all three metabolic diseases and the comorbidities, clinical cohort studies have shown the consistent depletion of the genera *Eubacterium, Roseburia* and *Feacalibacterium* in diseased patients. Notably, these are all butyrate producing bacteria. This important short chain fatty acid plays a key role in maintaining health and inhibiting inflammation [55]. Other genera, including *Bacteroides*, *Coprococcus* and *Ruminococcus* showed inconsistent trends across different diseases. *Bacteroides* enterotype is associated with long-term diet choices, usually high protein [56]. It is well known the relationship between diet and the microbiome can contribute to the development of disease. However, when reviewing this literature, it is key that we interpret their findings with caution. Each of these studies used different designs and sequencing techniques as well as other variations in the data, such as geographical region or age of the participants. These are all factors which, as stated above, are known to impact on both microbiome and on the disease phenotype itself.

## 3. Alterations in the Microbiome Associated with Medical Intervention or Treatment 

Much like the gut microbiome is associated with disease progression and phenotype it is increasingly being implicated in drug pharmacology and drug mechanism. Microbial consortia and function can be altered due to the impact of drugs or other interventions, such as low-calorie diets, exercise regimes or surgery [57,58]. Natural products such as probiotics, prebiotics and synbiotics (such as sauerkraut, kimchi and live yoghurt) can also affect the microbiome [59]. These products are often considered beneficial for gut health. Here, we specifically review commonly used medical interventions with acknowledged clinical effectiveness and hence is the best known treatment derived from research and clinical experience to achieve optimum outcome. As such, although likely effective, natural products fall out of the scope of this review. 

The extent of drug-induced alterations made to the gut microbiome are unique to every person, down to the compositional changes at strain-level [58]. Additionally, they noted the potential role microbes play in metabolising drugs [58]. Therefore, we must start considering the gut microbiome as a key factor in drug therapy as well as in disease [60]. Some drugs specifically target bacteria (such as antibiotics), whilst the mechanism of others such as increased production of short chain fatty acids (SCFAs) is linked with the microbiome (such as metformin) and others alter the microbial composition (such as PPIs). However, the effect these drugs have on the microbiome is not considered when prescribing them. Recently, studies have started looking at the impact drugs or interventions have on the gut microbiome (Supplementary Table S2). 

### 3.1. Alterations in the Gut Microbiome Due to Diet and Exercise Interventions

Obesity and T2D are both predominantly caused by excessive food intake and lack of exercise [61]. Hence, the first line of treatment for obesity is usually a diet resulting in a calorific intake deficit and increased exercise. Diet-induced weight-loss interventions appear to improve microbiome composition and clinical phenotypes [62]. Several studies have exposed differences between the composition of lean versus obese subjects, and many groups have reported changes to lifestyle impacts the gut microbiome composition [63]. Metagenomic studies have now started to examine obese or overweight patients to examine the functional impact the changes to the microbiome have on the disease [62,64]. In T2D, low grade inflammation caused by poor diet is considered responsible for insulin resistance [65]. There is robust evidence for exercise treating or preventing T2D, and interestingly there is also evidence of regular exercise influencing the gut microbiome composition [64,66]. However, it is currently unclear whether this influence is on the microbes directly or via the impact exercise has on the host. To investigate these lifestyle alterations, studies have put obese patients on a low-calorie diet and exercise therapy over 24 months [64] or T2D patients through a 6-month exercise program [13]. Metagenomic analyses of stool from these cohorts at baseline and study-end showed an increased abundance in Proteobacteria with both interventions. However, increased abundance of Proteobacteria has also been associated with metabolic diseases and been identified as a potential microbial signature of disease. Hence, it is possible that whilst these interventions treated the symptoms of the diseases, their impact on the microbiome was not as positive.

### 3.2. Alterations in the Gut Microbiome Due to Metformin

After lifestyle intervention, metformin is the first drug used to treat T2D where it acts as an antihyperglycemic [67], supressing hepatic gluconeogenesis [68] and increases skeletal muscle tissue glucose uptake [69]. However, to date, the complete mechanism of action of metformin is still unknown. There is mounting evidence to suggest that the drug interacts with the gastrointestinal tract and studies have shown that metformin treatment also has a significant impact on the microbiome [70,71,72]. E. H. Ejtahed et al. [71] noted the weight loss seen in diabetics treated with metformin, and hence studied the impact of metformin treatment in non-diabetic obese patients on their gut microbiome composition. They showed treatment with metformin caused significant weight-loss in conjunction with distinct differences in taxonomy between baseline and post treatment. Concordant results from both studies showed increases in the phylum Proteobacteria including *Escherichia*, *Pseudomonas*, *Shigella* and *Yersinia*. 

### 3.3. Alterations in the Gut Microbiome Due to Cardiovascular Drugs

T2D and obesity are both often comorbidities of cardiovascular disease. ACVD is a cardiovascular disease which affects arterial walls [73]. Atherosclerotic plaques restrict blood flow through the coronary arteries. This plaque build-up is often caused by environmental factors such as high-fat diet, smoking and lack of exercise. Hence, it is not surprising the recommended treatment for ACVD when first diagnosed is increased exercise and following a healthy diet. Medication is often used to manage the symptoms of ACVD and prevent the disease from worsening. Statins are widely used for the treatment of high cholesterol [74]. These drugs promote the reduction in levels of LDL cholesterol [75] and can have anti-inflammatory effects [76]. Vieira-Silva et al. noted that a microbial composition associated with obesity was negatively associated with statin use. They also commented on the potential of statin treatment in the management of microbial dysbiosis in the gut by improving the inflammation status of the host [77]. Statins are not the only drugs used in the treatment of ACVD symptoms. Angiotensin-converting-enzyme (ACE) inhibitors treat high blood pressure and heart failure whilst *β*-blockers manage abnormal heart rhythms that occur in ACVD. Whilst PPIs are not used directly for cardiovascular complaints, they are often taken in conjunction with medication such as antiplatelets or aspirin as these drugs can cause indigestion and heartburn. PPIs are known to impact the microbiome [78,79] by the inhibiting secretion of gastric acid which increases the number of surviving microbes that pass through the stomach to reach the intestine [80]. In doing so, PPIs significantly alter the gut microbiome, with an increase in Actinobacteria at the phylum level, including *Bifidobacterium*, one of the most represented genera of Actinobacteria in the human gut. Despite all these drugs having different mechanisms, they have notably similar effects on microbiome composition, all causing an increase in *Blautia* and *Streptococcus* for example (Figure 1B) [78]. This could be due to the drugs having comparable functional effects on the microbial pathways, hence causing analogous outcomes in taxonomic profiles.

### 3.4. Alterations in the Microbiome after Gastric Band Surgery

Although dietary change and increased exercise is the standard recommended treatment for obesity, the results are varied predominantly due to variations in patient compliance. In contrast, bariatric surgical intervention is currently the most effective treatment for obesity; for example, patients who also suffer from T2D go into remission post intervention [26,81,82]. This surgical intervention could result in rapid weight loss, improved glucose metabolism and improved insulin sensitivity [83]. The three most common surgeries are laparoscopic adjustable Gastric band [84]; vertical sleeve gastrectomy (VBG) [26] and a gastric roux-en-y bypass (RYGB) [82,85]. Studies have indicated that gastric band surgery has beneficial effects on the composition of the gut microbiome [86]. These surgery-induced changes are usually associated with metabolic improvements. However, it is important to note these changes could be due to the decreased capacity of food intake or different dietary regime. Comparing the composition of the microbiome pre- and post-surgery with other interventions, there is a large overlap in those genera seen to be significantly affected by the surgery (Figure 1C) [26,82,83,84,85,86,87]. Many studies investigated the impact of the roux-en-y surgery on the gut microbiome [26,82,83,84,85,86,87]. RYGBs clinical cohort studies showed many genera in Actinobacteria and Proteobacteria were increased after surgery (Supplementary Table S3). There are clearer differences in microbial composition in those who underwent RYGB surgery compared to those who had VBG surgery, when compared to the patients’ pre-surgery microbiome [26]. Tremaroli et al. determined *Escherichia*, *Klebsiella* and *Pseudomonas* (all from Proteobacteria) were increased after RYGB, similar to the exercise and diet interventions mentioned earlier [26]. However, despite the differences between procedures they did not observe a significant difference in the microbial profiles between the two patient cohorts.

## 4. Metabolomic Signatures in Metabolic Diseases

**Figure 2:**
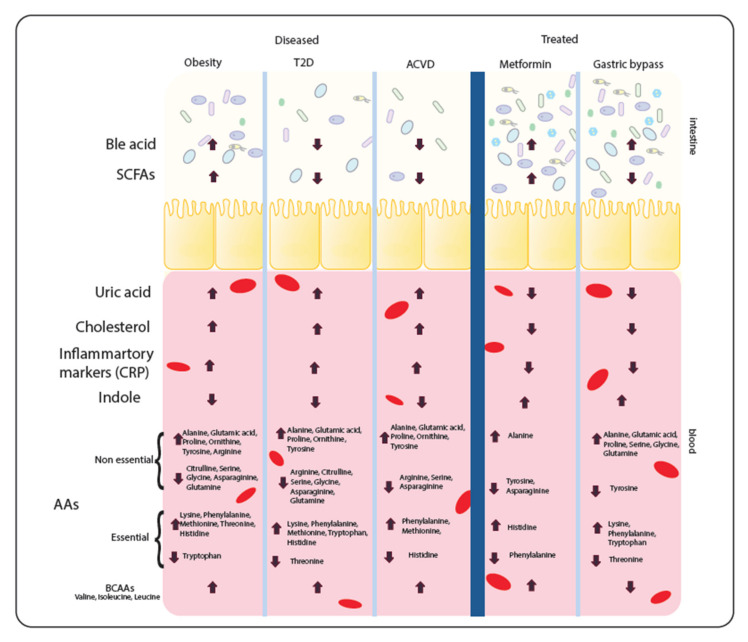
Metabolite levels seen in diseases and interventions. Dysbiosis in the intestine of metabolic disease patients causes alterations to the metabolite levels reported in faecal and serum samples. After treatment, the composition of the microbiome is altered again and hence the metabolite levels alter again. Here, specific metabolite levels which have been reported in metabolic disease (obesity, type 2 diabetes (T2D) and atherosclerosis (ACVD)) and the levels reported after metformin treatment or gastric band surgery. These are seen in the gut from stool samples or in the blood from serum/plasma samples. Metabolites noted with a down arrow are found to be decreased in the disease or treatment. Metabolites noted with an up arrow are found to be increased in the disease or treatment. Both an up and down arrow shows the metabolites vary in enrichment.

The symbiotic metabolic relationship between host and microbiome is reflected not just in the taxonomical signature of the microbiome, but also in the integrated metabolism between host and microbiome. This integrated metabolism will affect metabolite levels present in both gut/stool and blood. As a result, these metabolites as well as providing potential biomarkers for disease may also affect the disease progression itself. Reported levels of metabolites in metabolic diseases and common interventions are shown in Figure 2. Notably, the parallels seen in the metabolite profiles between the diseases indicates that metabolic disease similarities do not end at the level of microbiota dysbiosis. 

Metabolomic studies, comparing the small molecules and compounds found in the cells, tissues or biofluids of the host, have been performed across the different metabolic diseases. Despite different metabolomic approaches and obvious differences between the diseases, studies show a series of striking similarities in faecal and serum metabolite levels. In particular, there are consistent changes in serum levels of metabolites such as uric acid, cholesterol and inflammatory markers (including C-reactive protein metabolite (CRPM)), all common disease biomarkers. High levels of these metabolites (especially cholesterol and uric acid) in the bloodstream cause an inflammatory response, thus potentially driving disease progression. Thus, unsurprisingly, uric acid, cholesterol and inflammatory markers were all increased in metabolic diseases [88,89,90,91,92,93,94].

Serum metabolomics also showed depletion of indoles in all metabolic diseases [95,96]. Indoles are derived from tryptophan and are ligands for aryl hydrocarbon receptor (AHR), which in turn has an anti-inflammatory effect and stimulates γδ T-cell development [97,98]. However, there was no correlation between serum amino acids and disease, except branch chain amino acids (BCAAs) which were increased in abundance in disease [99,100,101,102]. Potentially, elevated BCAA serum levels could therefore be a new biomarker for metabolic disease. The faecal metabolome also showed several common themes. SCFAs such as acetate, butyrate and propionate were seen to be decreased in both T2D and ACVD [89,103], whilst obesity has increased levels of SCFAs [104]. Bile acids, composed of cholesterol metabolites and serving as metabolic regulators of glucose, fat and energy metabolism [105] were reported increased in obesity [106], while T2D and ACVD showed decreased levels [18,107]. Bile acids can be beneficial and detrimental to human health, hence an imbalance in bile acids could exacerbate metabolic disease [108].

Post-medical intervention usually drives a shift in metabolite levels. Uric acid, cholesterol and inflammatory markers all show a decrease after metformin treatment or gastric band surgery [92,109,110,111,112] whilst indole levels increase, providing a contrasting metabolite profile to untreated cohorts [95,113]. Additionally, bile acids were increased post-intervention compared to untreated cohorts [114,115]. Faecal SCFAs show varying responses from interventions [26,95]. Metformin increased levels, as expected, whilst SCFA levels were decreased in T2D. On the other hand, GBs decrease the SCFA levels to counter the increased levels seen in obesity. Amino acids again showed no correlation with intervention. Notably, however, BCAAs were decreased after GB surgery [116,117]. The metabolite levels are dictated by the changing microbiome as much as by the host, as different species produce and consume different compounds. For example, *Eubacterium* (decreased in all diseases) has a negative correlation with cholesterol and inflammatory markers [118]. To understand these links more clearly, it is important to consider the metabolism of the species understand their metabolic interaction between each other and host to elucidate their role in the disease. 

## 5. A Systems Biology Approach to Further Understanding Disease

**Figure 3:**
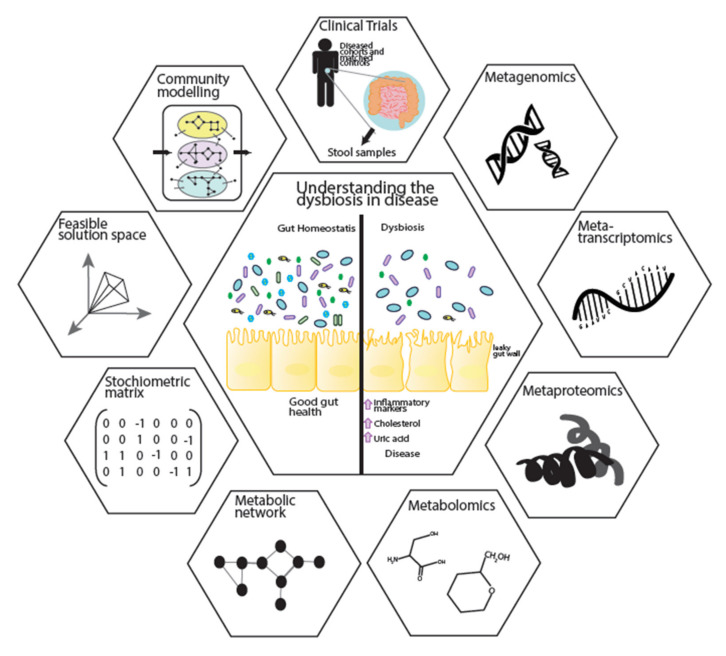
Application of GEMs in multi-omics data integration and ecosystem modelling. Clinical samples are taken from cohorts to generate multi-omics data, including metagenomics, metatranscriptomics, metaproteomics and metabolomics. Multi-omics data is used in the reconstruction of metabolic models. Constraint-based analysis can be applied on individual metabolic models to understand the metabolic capability of an organism. Following form individual modelling, community models can be created to show microbe–microbe and microbe–host interactions.

Metagenomics and metabolomics studies have revealed the associations of microbial abundance and the metabolite profile to metabolic diseases. However, these associations are mediated by complex interactions between the microbes, host and environmental factors such as diet. Systems biology, with its holistic view of biological systems, integrates these multi-omics data sets. They allow for the development of mathematical models that can be applied in microbial ecosystems to mechanistically reveal the role of individual bacteria and their interactions in disease progression (Figure 3). This integrative approach provides significant insights into biological mechanisms [119]. The centre of this integration can be biological networks, including metabolic networks, signalling networks, protein–protein interactions, co-expression networks and gene regulatory networks [120,121,122]. Signalling networks provide frameworks for dynamic and static modelling of cell signalling, while protein–protein interaction networks give insights to the interaction of proteins which are integral to biological functions. Gene regulatory networks represent the link between transcription factors and genes. Among different network analyses, genome-scale metabolic models (GEM) describe the gene–protein–reaction relationships within an organism through collection of the various metabolic reactions, their stoichiometry information and compartmentalisation [120]. GEMs can be integrated with other biological networks to provide a more comprehensive view of the cellular behaviours.

Static and dynamic algorithms are commonly applied for modelling of biological networks such as kinetic, petri-net, stoichiometric, hybrid approaches and structural modelling [123]. Constraint-based modelling can also be applied to GEMs by introducing specific constrains (such as input substrates or gene expression data) to optimise an objective function (the reaction desired to be maximised or minimised). In most microbial cases biomass is the objective function used to predict cellular metabolism and biochemical production for both catabolic and anabolic processes [124]. Additionally, all the information needed for GEM reconstruction is a list of biochemical reactions and their stoichiometry matrix. With these unique capabilities, GEMs link genotype to phenotype, underlining the mechanism behind different conditions, such as cancer, metabolic disease or liver disease. In doing so, they identify potential new biomarkers and therapeutic interventions [121,125,126]. Recently, GEMs have been applied to microbial ecosystem. To do this GEMs for several species have been reconstructed [127] and applied to community-level analysis [120]. GEMs can be applied to understand the interaction between microbes by introducing the biomass as the individual objective functions governing the optimisation of the microbial ecosystems’ growth as the community objective [128,129]. This allows us to investigate in silico how microbes respond to environmental alterations such as diet interventions, meaning we can quantitatively describe the change in the uptake or secretion of metabolites resulting from any intervention [130]. By combining microbial GEMs with a comprehensive host metabolic model, such as Recon 3D [131] the metabolic interactions between the microbial community and host can be studied [132]. This then opens the door to personalised community modelling that can be used for microbe–microbe interactions [133]. Thus, GEMs represent a powerful way forward to elucidate the causality of the microbial community changes to their associated diseases, revealing their metabolic role in both the gut and the wider host habitat. 

## 6. Conclusions 

Despite having identified microbial association across the metabolic diseases, there is still a mechanistic understanding missing of how microbe–host interaction changes contribute to disease pathophysiology. Accumulating evidence indicates that these diseases are associated with each other and also have strong bidirectional links to the microbiome. Obesity, insulin resistance or cardiovascular disease are often co-morbidities and are increasingly seen with other diseases. Furthermore, the recommended treatments for these diseases do not simply impact the symptoms and host physiology—they also result in alterations to the gut microbiome. From a future perspective, there is a need for further studies and data generation of the alterations in the microbiome in all diseases. Additionally, clearly the effects drugs have on the microbiome should be considered in the risk assessment of new drugs, or should be considered as a potential target for medical interventions. The microbiome could be used in a novel method of attaining biomarkers for the progression of diseases. By targeting the microbiome directly, either through composition or metabolism, we can open up new avenues for managing these chronic, debilitating diseases most effectively.

## Supplementary Materials

The following are available online at https://www.mdpi.com/2076-2607/8/9/1381/s1, Table S1: Bacterial taxa increased and decreased in diseases. (N) is the number of studies data was taken from. Bold—genera that were reported in more than one study. Table S2: Bacterial taxa increased and decreased in medical interventions or after a course of medical drug treatment. (N) is the number of studies data was taken from. Bold—genera that were reported in more than one study. Table S3: Bacterial taxa increased and decreased after gastric band surgery. (N) is the number of studies data was taken from. Bold—genera that were reported in more than one study.
